# Supplementary material for: Allosteric regulation and crystallographic fragment screening of SARS-CoV-2 NSP15 endoribonuclease
Source: Nucleic Acids Res. 2023 Apr 28;51(10):5255–70. doi: 10.1093/nar/gkad314 (PMC10250223; doi:10.1093/nar/gkad314)
Supplement: gkad314_Supplemental_Files [file gkad314_supplemental_files.zip › Supplementary datasets.zip!/139/Refine_1/01-BUSTER/Cycle-3/shell.01/R_Rfree.html]

# Rxpct factors in resolution bins at cycle 0


|  |  |  |  |  |  |  |  |  |
| --- | --- | --- | --- | --- | --- | --- | --- | --- |
|  | **<d> (Å)**  Rxpct\_Work | Rxpct\_Free | Rxpct\_All | |  |  |  |  |
|  | 13.2733 | 0.3183 | 0.4632 | 0.3244 |  |
|  | 7.6634 | 0.2571 | 0.2524 | 0.2568 |  |
|  | 5.9360 | 0.2821 | 0.2932 | 0.2826 |  |
|  | 5.0168 | 0.2286 | 0.2364 | 0.2291 |  |
|  | 4.4244 | 0.1997 | 0.2195 | 0.2007 |  |
|  | 4.0021 | 0.1941 | 0.1945 | 0.1942 |  |
|  | 3.6814 | 0.2030 | 0.2455 | 0.2051 |  |
|  | 3.4272 | 0.2068 | 0.2130 | 0.2072 |  |
|  | 3.2193 | 0.2009 | 0.2247 | 0.2022 |  |
|  | 3.0451 | 0.2017 | 0.2129 | 0.2022 |  |
|  | 2.8965 | 0.2069 | 0.2233 | 0.2076 |  |
|  | 2.7677 | 0.2028 | 0.2207 | 0.2036 |  |
|  | 2.6547 | 0.2092 | 0.2319 | 0.2106 |  |
|  | 2.5545 | 0.2016 | 0.2287 | 0.2031 |  |
|  | 2.4648 | 0.1996 | 0.2195 | 0.2005 |  |
|  | 2.3840 | 0.1995 | 0.2303 | 0.2010 |  |
|  | 2.3106 | 0.1949 | 0.1865 | 0.1946 |  |
|  | 2.2436 | 0.1952 | 0.2066 | 0.1958 |  |
|  | 2.1821 | 0.1884 | 0.1777 | 0.1879 |  |
|  | 2.1254 | 0.1932 | 0.1916 | 0.1932 |  |
|  | **Overall** | 0.2158 | 0.2340 | 0.2168 |  |

# Rxpct factors in resolution bins at cycle 1


|  |  |  |  |  |  |  |  |  |
| --- | --- | --- | --- | --- | --- | --- | --- | --- |
|  | **<d> (Å)**  Rxpct\_Work | Rxpct\_Free | Rxpct\_All | |  |  |  |  |
|  | 13.2733 | 0.3199 | 0.4668 | 0.3261 |  |
|  | 7.6634 | 0.2586 | 0.2549 | 0.2584 |  |
|  | 5.9360 | 0.2828 | 0.2937 | 0.2832 |  |
|  | 5.0168 | 0.2281 | 0.2368 | 0.2286 |  |
|  | 4.4244 | 0.1993 | 0.2192 | 0.2004 |  |
|  | 4.0021 | 0.1936 | 0.1948 | 0.1937 |  |
|  | 3.6814 | 0.2029 | 0.2443 | 0.2048 |  |
|  | 3.4272 | 0.2066 | 0.2131 | 0.2070 |  |
|  | 3.2193 | 0.2002 | 0.2224 | 0.2014 |  |
|  | 3.0451 | 0.2017 | 0.2131 | 0.2022 |  |
|  | 2.8965 | 0.2060 | 0.2229 | 0.2067 |  |
|  | 2.7677 | 0.2028 | 0.2166 | 0.2034 |  |
|  | 2.6547 | 0.2091 | 0.2303 | 0.2104 |  |
|  | 2.5545 | 0.2016 | 0.2290 | 0.2032 |  |
|  | 2.4648 | 0.1995 | 0.2204 | 0.2005 |  |
|  | 2.3840 | 0.1998 | 0.2338 | 0.2014 |  |
|  | 2.3106 | 0.1955 | 0.1860 | 0.1951 |  |
|  | 2.2436 | 0.1955 | 0.2038 | 0.1959 |  |
|  | 2.1821 | 0.1893 | 0.1779 | 0.1888 |  |
|  | 2.1254 | 0.1945 | 0.1922 | 0.1944 |  |
|  | **Overall** | 0.2159 | 0.2340 | 0.2168 |  |

# Rxpct factors in resolution bins at cycle 2


|  |  |  |  |  |  |  |  |  |
| --- | --- | --- | --- | --- | --- | --- | --- | --- |
|  | **<d> (Å)**  Rxpct\_Work | Rxpct\_Free | Rxpct\_All | |  |  |  |  |
|  | 13.2733 | 0.3211 | 0.4695 | 0.3274 |  |
|  | 7.6634 | 0.2602 | 0.2582 | 0.2601 |  |
|  | 5.9360 | 0.2841 | 0.2954 | 0.2845 |  |
|  | 5.0168 | 0.2285 | 0.2389 | 0.2292 |  |
|  | 4.4244 | 0.1992 | 0.2202 | 0.2002 |  |
|  | 4.0021 | 0.1928 | 0.1950 | 0.1929 |  |
|  | 3.6814 | 0.2026 | 0.2447 | 0.2046 |  |
|  | 3.4272 | 0.2068 | 0.2134 | 0.2072 |  |
|  | 3.2193 | 0.2003 | 0.2231 | 0.2016 |  |
|  | 3.0451 | 0.2018 | 0.2133 | 0.2023 |  |
|  | 2.8965 | 0.2059 | 0.2242 | 0.2067 |  |
|  | 2.7677 | 0.2034 | 0.2178 | 0.2041 |  |
|  | 2.6547 | 0.2099 | 0.2305 | 0.2112 |  |
|  | 2.5545 | 0.2020 | 0.2297 | 0.2036 |  |
|  | 2.4648 | 0.1997 | 0.2228 | 0.2008 |  |
|  | 2.3840 | 0.1998 | 0.2323 | 0.2013 |  |
|  | 2.3106 | 0.1966 | 0.1846 | 0.1960 |  |
|  | 2.2436 | 0.1965 | 0.2044 | 0.1969 |  |
|  | 2.1821 | 0.1900 | 0.1788 | 0.1895 |  |
|  | 2.1254 | 0.1952 | 0.1921 | 0.1950 |  |
|  | **Overall** | 0.2162 | 0.2349 | 0.2171 |  |

# Rxpct factors in resolution bins at cycle 3


|  |  |  |  |  |  |  |  |  |
| --- | --- | --- | --- | --- | --- | --- | --- | --- |
|  | **<d> (Å)**  Rxpct\_Work | Rxpct\_Free | Rxpct\_All | |  |  |  |  |
|  | 13.2733 | 0.3150 | 0.4605 | 0.3211 |  |
|  | 7.6634 | 0.2549 | 0.2477 | 0.2544 |  |
|  | 5.9360 | 0.2713 | 0.2775 | 0.2716 |  |
|  | 5.0168 | 0.2211 | 0.2330 | 0.2218 |  |
|  | 4.4244 | 0.1957 | 0.2184 | 0.1969 |  |
|  | 4.0021 | 0.1925 | 0.1983 | 0.1928 |  |
|  | 3.6814 | 0.2012 | 0.2423 | 0.2032 |  |
|  | 3.4272 | 0.2053 | 0.2151 | 0.2059 |  |
|  | 3.2193 | 0.1980 | 0.2292 | 0.1997 |  |
|  | 3.0451 | 0.1982 | 0.2100 | 0.1988 |  |
|  | 2.8965 | 0.2019 | 0.2203 | 0.2027 |  |
|  | 2.7677 | 0.1990 | 0.2136 | 0.1996 |  |
|  | 2.6547 | 0.2061 | 0.2203 | 0.2069 |  |
|  | 2.5545 | 0.1993 | 0.2245 | 0.2007 |  |
|  | 2.4648 | 0.1962 | 0.2209 | 0.1974 |  |
|  | 2.3840 | 0.1984 | 0.2303 | 0.1999 |  |
|  | 2.3106 | 0.1931 | 0.1843 | 0.1927 |  |
|  | 2.2436 | 0.1955 | 0.2048 | 0.1960 |  |
|  | 2.1821 | 0.1891 | 0.1804 | 0.1887 |  |
|  | 2.1254 | 0.1957 | 0.1921 | 0.1955 |  |
|  | **Overall** | 0.2126 | 0.2319 | 0.2135 |  |
